# Exploring the Role of CYP3A4 Mediated Drug Metabolism in the Pharmacological Modulation of Nitric Oxide Production

**DOI:** 10.3389/fphar.2017.00202

**Published:** 2017-04-12

**Authors:** José Pérez-del Palacio, Caridad Díaz, Noemí Vergara, Francesca Algieri, Alba Rodríguez-Nogales, Nuria de Pedro, M. Elena Rodríguez-Cabezas, Olga Genilloud, Julio Gálvez, Francisca Vicente

**Affiliations:** ^1^Fundación MEDINA, Centro de Excelencia en Investigación de Medicamentos Innovadores de AndalucíaGranada, Spain; ^2^Calcium Metabolism and Vascular Calcification Unit, Maimonides Institute for Biomedical Research, University Hospital Reina Sofia, Nephrology Service, University of CórdobaCordoba, Spain; ^3^Department of Pharmacology, ibs, CIBER-EHD, Center for Biomedical Research, University of GranadaGranada, Spain

**Keywords:** nitric, oxide CYP450, drug metabolism, immunomodulation

## Abstract

Nitric-oxide synthase, the enzyme responsible for mammalian nitric oxide generation, and cytochrome P450, the major enzymes involved in drug metabolism, share striking similarities. Therefore, it makes sense that cytochrome P450 drug mediated biotransformations might play an important role in the pharmacological modulation of nitric oxide synthase. In this work, we have undertaken an integrated *in vitro* assessment of the hepatic metabolism and nitric oxide modulation of previously described dual inhibitors (imidazoles and macrolides) of these enzymes in order assess the implication of CYP450 activities over production of nitric oxide. *In vitro* systems based in human liver microsomes and activated mouse macrophages were developed for these purposes. Additionally *in vitro* production the hepatic metabolites of dual inhibitor, roxithromycin, was investigated achieving the identification and isolation of main hepatic biotransformation products. Our results suggested that for some macrolide compounds, the cytochrome P450 3A4 derived drug metabolites have an important effect on nitric oxide production and might critically contribute to the pharmacological immunomodulatory activity observed.

## Introduction

In a broad sense, nitric-oxide synthase (NOS) belongs to the CY450 family since the label cytochrome P450 (CYP450) has come to encompass a huge and widely distributed group of closely related enzymes containing a thiolate-ligated heme. Although structurally different, they have a mechanistic resemblance since NOS converts L-arginine to L-citrulline and nitric oxide (NO) through a P450-like process and complementarily CYP450 was also found able to transform N-hydroxy-L-arginine into NO and citrulline. Thus, the first step in NO biosynthesis appears to have some precedence in P450 chemistry (Gorren and Mayer, 2007). Perhaps most striking among the similarities between CYP450 and NOS is the fact that both the NOS oxygenase domain and mammalian CYP450 utilize the same electron donor as a redox partner (Masters, 2005). Given the extensive similarity between NOS and CYP450 and the vast repertoire of drug P450-catalyzed biotransformations (Bredt et al., 1991; Sono et al., 1996; Meunier et al., 2004), it is worth assessing the role CYP450 might play in the development of drug immunomodulation. Therefore, we consider that molecules showing ability to inhibit NOS activity might be especially prone to interact with CYP450 family. The CYP450 is the main metabolic systems to deal with lipid-soluble environmental chemicals and consequently it plays a critical role in drug metabolism. The CYP450 system performs this function by oxidizing, hydrolyzing or reducing the chemicals. This enables another group of enzymes, conjugation enzymes, to attach polar groups to make the metabolites water soluble so that they can be excreted in the urine (Martin and Fay, 2001). Drug metabolism might results in the production of both inactive and active metabolites. In fact, active metabolites might be more potent than the parent compound. Thus, although metabolism is ultimately a process of detoxification, it produces intermediate products that may have clinically useful activity, be associated with toxicity, or both (Smith, 2009). Therefore the biological effectiveness and the potential toxicity of NOS inhibitors might be strongly influenced by their CYP450 metabolism.

Due to the association between the inappropriate release of NO and the pathogenesis of a number of disease states, the NOS isoforms (mainly the inducible isoform, iNOS) have become attractive targets in drug design for various pathological states, especially in neurodegenerative disorders, inflammation and pain (Joubert and Malan, 2011).

Among known compounds able to prevent the biological activity of NOS, we can find the antifungal imidazole derivate family which is also recognized for strongly inhibiting the enzymatic activity of several CYP450 isoforms (Niwa et al., 2005). This inhibition of the NOS involves a putative binding of the imidazole nitrogen to the heme iron, reducing the maximal velocity of citrulline formation (Wolff et al., 1993). Not surprisingly, in other mechanistic studies for evaluation of CYP3A4 inhibition by imidazole derivatives, heme coordination by the imidazole functional group was observed (Hutzler et al., 2006). Analogously, macrolide antibiotics have long been recognized to exert immunomodulatory and anti-inflammatory actions, via inactivation of NO production (Gao et al., 2008; Buret, 2010). In turn, some macrolide antibiotics have also been found to form stable metabolic intermediate complexes with the iron (II) present in the heme group of CYP450, after being metabolized (Yamazaki and Shimada, 1998; Zweers-Zeilmaker et al., 1999). The main objective of this work was to assess *in vitro*, the implication of the CYP450 activities in the modulation of NO production. In this regard we proposed a working hypothesis in which well-known compounds (imidazole derivatives & macrolides) with the ability for inhibiting both activities (CYP450 and NOS) might display differential ability to inhibit NO production depending on the CYP450 functionality of the cell *in vitro* model considered. It has been extensively reported that expression of biotranforming enzymes in immortalized cell lines is much lower on average than that observed in primary cells from liver, lung, blood, and skin (Soldatow et al., 2013). Therefore if CYP450 activities are modulating to some extent NO production by compound depletion or bioactive metabolite formation, some differential effects in NO production should be observed between *in vitro* models using cell lines or primary cultures. To this end, we used an immunocompetent *in vitro* model, but with limited CYP450 functionality, based on lipopolysaccharide (LPS) stimulated, cultured cell lines of murine macrophages (RAW 264.7) (Rodríguez-Antona et al., 2002; Garrido-Mesa et al., 2010). These results were compared with those retrieved from literature using primary cultures of alveolar macrophages in which the pattern of CYP450 expression was found to closely resemble the expression pattern in lung tissue (Hukkanen, 2000; Hukkanen et al., 2002). In order to correlate NO measurements with CYP450 activity, we also assessed the before mentioned dual inhibitors in classical *in vitro* models for drug metabolism assessment such as CYP450 reversible and time-dependent inhibition, aqueous solubility and microsomal stability with metabolite identification (Obach, 1999; Obach et al., 2006; Burt et al., 2010; Yang et al., 2014; Perez et al., 2015). These *in vitro* studies using microsomal fractions have historically provided critical and basic information on drug metabolism by CYP450 which is essential for prediction of the *in vivo* state (Cederbaum, 2015).

## Methods and materials

### Reagents

Fetal bovine serum (FBS), L-glutamine, sodium pyruvate, MEM non-essential amino acids, penicillin–streptomycin, and TrypLE Express were purchased from Invitrogen Gibco, Inc. (Life Technologies, Carlsbad, CA). NP40 was purchased from Thermo Scientific (Rockford, IL). Diphenyl tetrazolium bromide (MTT), and methyl methane sulfonate (MMS) were obtained from Sigma Aldrich. The commercial compounds evaluated in this work, ketoconazole, miconazole, clotrimazole, erythromycin (ERY), roxythromycin (RXT), azythromycin (AZT), and clarithromycin (CLT), were obtained from Sigma Aldrich (St. Louis, MO). Human recombinant c-DNA expressed CYP3A4 at 1 nmol/mL was obtained from Gentest Corporation. Pooled human liver microsomes (HLM) were obtained from Becton Dickinson Gentest (Woburn, MA).

### Test compound preparation

For the simultaneous evaluation of CYP3A4 reversible inhibition and solubility using the NIVA-CYPI-KS (Perez et al., 2015), commercial compounds were prepared as follows: test compounds were provided in powder form and dissolved in 100% DMSO at 25 mM in 96-well plates. Serial dilutions of test compounds in 100% DMSO were carried out on a Biomek FX workstation coupled with a stacker carrousel (Beckman Coulter Inc. Brea, California). Considering their previously reported CYP3A4 inhibition potential, commercial compounds were prepared at different initial concentrations and dilution factors in order to optimize their IC_50_ calculation from a titration curve with 8 concentration levels. Due to the extensively reported ability of DMSO to inhibit CYP3A4 activity, diluted compounds in 100% DMSO (35 μL) were combined with AcN (65 μL) in 96-well microtiter plates (AB-0765, Thermo, Waltham, MA) by a Perkin Elmer Evolution P3 liquid-dispensing instrument (Waltham, Massachusetts) in order to minimize the final DMSO content (0.35%) in enzyme incubations. DMSO has been described as a potent inhibitor of several CYP450 isoforms (Chauret et al., 1998). Compounds for NO production assessment were also prepared from serial dilutions in 100% DMSO (20 μL) which were combined with water (80 μL). Initial concentrations and dilution patterns were also adjusted for each compound depending on the calculated solubility values and expected response in NO production related studies (macrolides and imidazole derivatives at ten concentration levels from 0.48 to 250 μM and from 0.019 to 10 μM, respectively).

### Assays for NO production assessment in LPS stimulated murine macrophages

The mouse macrophage cells, RAW264.7 (ATCC® TIB-71™) were obtained from ATCC and cultured in Dulbecco's Modified Eagle Medium (DMEM), supplemented with 10% FBS and 2 mM l-glutamine, in a humidified 5% CO_2_ atmosphere at 37°C. All cell handling steps were carried out using the SelecT automated cell culture system. RAW264.7 cells were seeded into 96-well plates at a density of 20 × 10^3^ cells per well and grown to approximately 50% of confluence (24 h). They were cultured for another 24 h with each of the test compounds described above (5 μL of stock serial dilutions in 20% DMSO were dispensed into 190 μL of fresh medium using Perkin Elmer Evolution P3, Waltham, MA,). Afterwards, these cells were stimulated with LPS (5 μL of 6 μg/mL were dispensed using a Thermo Scientific Multidrop Combi dispenser, MTX Lab Systems, Vienna, VA). Untreated (5 μL of 20% DMSO, compound vehicle) and unstimulated (5 μL of purified water, LPS vehicle) cells were used as negative controls (*n* = 4) while untreated but stimulated cells were used as positive controls (*n* = 4) in each assay plate. After 24 h, supernatants were collected and centrifuged at 10,000 × g for 5 min, and nitrite levels were measured in 100 μL of supernatant according to the Griess assay using a Thermo Scientific Multidrop Combi dispenser and PerkinElmer EnVision Multilabel Reader (Waltham, MA) (Green et al., 1982). After discarding the remaining supernatant, the cell viability was examined in the remaining cells through the MTT test described elsewhere and also using a Thermo Scientific Multidrop Combi dispenser and PerkinElmer EnVision Multilabel Reader (Mosmann, 1983). Those compound concentrations showing cell viability <80% were rejected for further calculations.

#### Data analysis

Test compound activities were calculated automatically using the Genedata Screener software (Genedata AG, Basel, Switzerland), and the percentage inhibition of each commercial compound was determined by Equation (1), integrated in the Genedata Screener software. Percentage of control of production of nitric oxide was:

(1) $$Percentage\;of\;control\;=\;\left(\frac{Abs_{well}\;-\;Abs_{neg}}{Abs_{pos}\;-\;Abs_{neg}}\right)\;*\;100$$

where *Abs*_*well*_ is the absorbance measured per specific well; and *Abs*_*pos*_ and *Abs*_*neg*_ are the average absorbance measured for the positive (LPS/solvent vehicle) and negative controls (-LPS/solvent vehicle, respectively). IC_50_ values were calculated as the compound concentration that inhibits the production of NO by 50% using Genedata Screener software from dose-response curves in the above described assays (0.5% DMSO concentration). Dexamethasone was used as positive control standard. All compounds were tested in triplicate (*n* = 3) in two different days.

#### Statistical tests

Data replicates are presented as mean values ± STDEV. The coefficient of variation (relative STDEV) of each set of replicate data was calculated. A set of replicate date was considered acceptable if the coefficient of variation was ≤ 20 %.

### Drug metabolism assessment

#### Simultaneous CYP3A4 reversible inhibition and turbidimetric solubility evaluation (NIVA-CYPI-KS)

In this work, we used a novel *in vitro* approach (NIVA-CYPI-KS) to simultaneously assess CYP450 inhibition and aqueous compound solubility as previously described (Perez et al., 2015). Briefly, CYP3A4 inhibition studies were conducted using human recombinant c-DNA expressed CYP3A4 enzymes by quantification of non-fluorescent substrate, 7-benzyloxy-4-(trifluoromethyl) coumarin (BFC) transformation to a fluorescent product (7-HFC), which is mediated by CYP3A4. For aqueous solubility assessment, serial dilutions of test compounds (imidazole derivatives and macrolides) dissolved in DMSO/AcN [35:65] [v/v] (2 μL) were combined with a 180 μL potassium phosphate buffer (pH 7.4) using an Evolution P3 liquid handling station. Then the plates were incubated for 2 h at 37°C to allow slow compound precipitation. Thereafter, absorbance was measured at 620 nm by an EnVision multilabel plate reader. Absorbance signals were used to determine compound solubility limits. Pyrene was used as standard control for precipitation in turbidimetric kinetic solubility determination. Next, CYP3A4 enzyme (25 pmol/mL) and BFC (20 μM) were dispensed into the previous buffer/test compound solutions and reaction mixtures were incubated with shaking for 15 min at 37°C. Finally, reaction product (7-HFC) formation was quantify using florescence measurements. All compounds were tested in triplicate in two different days.

#### Assessment of CYP3A4 time-dependent inhibition

Experiments were designed according to previous works with minor modifications (Polasek and Miners, 2006). Briefly, pre-incubation mixtures contained human recombinant CYP3A4 (25 pmol/mL), NADPH-regenerating system (1 mM NADP, 10 mM glucose 6-phosphate, 2 IU/mL glucose 6-phosphate dehydrogenase, 5 mM MgCl_2_), test compound solutions (ERY, RXT, CLT, AZT, and ketoconazole) at least five different concentrations, and phosphate buffer (0.1 M, pH 7.4) in a total volume of 500 μL. At 0, 5, 10, 15, 20, 25, or 30 min, BFC (dissolved in AcN) was added so that the final concentration of BFC in the incubations was 25 μM. Incubations were allowed to proceed for a further 15 min at 37°C. Reactions were terminated with the addition of 75 μL of a STOP solution of AcN containing 0.5 M Tris base using a Multidrop liquid dispenser. Then fluorescence for 7-HFC formation was determined using 430 nm as excitation and 535 nm as emission wave lengths by an EnVision multilabel plate reader. Stock solutions of macrolides were prepared in DMSO/AcN as previously described and added to the pre-incubation mixtures so that the concentration ranges were: ERY, RXT, and AZT, 0.133 to 32.5 μM; CLT, 0.06 to 16.2 μM; ketoconazole, 0.0021 to 0.512 μM. The DMSO content in incubations was kept at 0.35% through all experiment. Control samples were prepared in the absence of test compounds by the addition of solvent alone.

##### Data analysis

The mean value of 7-HFC formation expressed as a percentage of control was used to estimate the kinetic constants of inactivation (K_inact_ and KI) according to related works (Silverman, 1995). Briefly, the observed inactivation rate constant at each macrolide concentration was calculated from the initial slopes of the remaining enzyme activity, plotted semi-logarithmically against the pre-incubation time. Non-linear regression analysis of the negative slopes against inhibitor concentration enables K_inact_ and KI to be calculated. Verapamil (tested at concentrations ranging from 0.279 to 68 μM) was used as positive control. All compounds were tested in triplicate in two different days for every methodology studied. All compounds were tested in a single experiment.

#### Simultaneous determination of *in vitro* metabolic profiling and metabolic stability

Test compounds (ERY, RXT, CLT, and AZT) were incubated in HLM at an initial substrate concentration of 1 μM in a 96-well format. Standard high-throughput incubation conditions were used with time points of 0, 15, 30, 45, 60, and 90 min. Protein concentration was 1 mg/mL and NADPH was present at 4 mM. Reaction was quenched using an equal volume of AcN and then diluted 1:1 with water prior to analysis by liquid chromatography coupled to high resolution mass spectrometry (LC-HRMS): **Chromatography**. The incubations were analyzed using an Agilent Series 1290 LC system (Agilent Technologies, Santa Clara, CA, USA). All analyses were performed using a Supelco Discovery HS C18 (2.1 × 50 mm) 3 μm column that was held at 30°C. Solvent A contained water with 0.1% formic acid and solvent B contained AcN with 0.1% formic acid, and the flow rate was set at 400 μL/min. The gradient elution was performed as follows: 0–0.5 min 0% eluent B; 0.5–7 min 100% eluent B; 7–9 min 100% eluent B; 9–9.2 min 0% eluent B; and 9.2–10.5 min 0% eluent B. **High resolution mass spectrometry**: AB SCIEX TripleTOF 5600 quadrupole-time-of-flight mass spectrometer (Q-TOF-MS) in positive ESI mode (AB SCIEX, Concord, ON, Canada) was used with a generic method for data acquisition on all compounds and samples. The method consisted of a time of flight mass survey scan (TOF MS), followed by two information-dependent acquisitions (IDA) TOF MS/MS scans. The mass range was m/z 100–1,000 for both MS and MS/MS. An accumulation time of 100 ms was used for each scan. Mass defect triggered IDA was used for the MS/MS scans (collision energy of 35 eV with a spread of ±10 eV). External mass calibration was performed automatically using a calibrant delivery system. Data processing was performed using MultiQuant Software and MetabolitePilot Software (AB SCIEX, Concord, ON, Canada) to process the TOF MS data and generate all quantitative information. An extracted ion current (XIC) window of ±10 mDa was used for all compounds.

#### Data analysis

Peak areas were used to plot the Ln % remaining relative to *t* = 0. The slope of the natural log of the percent remaining vs. time was calculated to determine the first-order rate constant (*k*) and the half-life (*T*_1/2_) of the test compounds according to Equation (2):

(2) $$Half\;life\;(T_{1/2})\;=\;0.693/k\;(\text{min})$$

Tentative metabolite identification was performed using MetabolitePilot Software (AB SCIEX, Concord, ON, Canada).for predicted and unpredicted metabolites, including fragment ion interpretation from precursor high resolution MS/MS spectra and potential cleavage metabolites. As a result a confirmation score (from 0 to 100) is provided to rank metabolite peak identification. In addition, formation rates of the metabolites were correlated to different incubation times to investigate metabolite stability. All compounds were tested in triplicate in two different days.

#### Statistical tests

Data replicates are presented as mean values ± STDEV. The coefficient of variation (relative STDEV) of each set of replicate data was calculated. A set of replicate date was considered acceptable if the confident of variation was ≤ 20.

### Production of RXT main hepatic metabolites

In order to provide a large enough amount of RXT main hepatic metabolites, large-scale human microsomal incubations were performed. Incubations were carried out in glass tubes containing HLM (2 mg of protein/mL), 5 mM MgCl_2_, 2 mM NADPH, 500 μM RXT, and 50 mM phosphate buffer (pH 7.4) in a total volume of 50 mL. After incubation in a 37°C water bath shaker for 240 min, the reactions were terminated by adding 50 mL of ice-cold AcN. The mixture was vortexed for 3 min and centrifuged at 3,750 rpm for 15 min. The supernatant was collected and dried under nitrogen gas flow. The residue was reconstituted with 100 mL of water. This concentrate was applied to a pre-conditioned Waters Oasis HLB solid phase extraction (SPE) 96-well plates (30 mg). The pre-conditioning step included washing with 1 mL each of neat methanol and 20% aqueous methanol. The loaded cartridge was washed with water, after which the metabolites were eluted using 1 mL of methanol. After evaporation of SPE elution and further reconstitution in 50 mL of water, liquid—liquid extraction (LLE) was also applied as an isolation step. The RXT metabolites extract was contacted with an organic solvent [(methyl—ethyl ketone), 1:1] and shaken vigorously for 10 min. Both, SPE and L/L procedures were optimized for achieving maximum recuperation of main RXT metabolites which had been previously described (Zhong et al., 2000). Centrifugation of the liquid mixture at 805 g for 15 min separated the organic layer from the aqueous layer. Samples from both layers were analyzed for metabolite content by LC-HRMS. The aqueous layer was dried under N_2_ current and further reconstituted in 5% aqueous methanol for subsequent preparative HPLC (high performance liquid chromatography) isolations which were performed using an Atlantis T3 column, 10 × 250 mm, 5-μm particle size. For isolation of metabolites, the mobile phase consisted of a linear gradient from 5 to 95% of mobile phase B (AcN containing 0.1% formic acid) in 25 min. The flow rate was 2.3 mL/min and UV absorbance was monitored at 290 nm. Fractions were automatically collected every 0.25 min and further analyzed by LC-HRMS. Each fraction was further analyzed by LC-HRMS using a fast gradient in 3.5 min in order to assess the metabolite purity and fraction enrichment. At this point it is important to comment that in parallel, we carried out blank (not containing RXT) large scale incubations that were equally subjected to SPE, LLE and semi-preparative HPLC fractionation in order to be used as false positive controls in the biological activity assessment.

## Results

### Effect of dual inhibitors on NO oxide production and correlated measurements of solubility and CYP3A4 inhibition

The results from the LPS stimulated RAW 264.7 macrophages *in vitro* model showed that the 3 members of the imidazole derivate family displayed moderate to weak inhibition potential in NO production (IC_50_ = 7.20, >10 μM and 7.35 μM, respectively, Table 1), in good agreement with previous works using cultured cell line models (Bogle and Vallance, 1996). However and based on their demonstrated ability to interact with the heme moiety, we had expected a more intense potential to *in vitro* inhibit NOS. Our experiments for the parallel determination of CYP3A4 inhibition using isolated enzymes and aqueous solubility confirmed the strong potential of these compounds to inhibit the enzymatic activity (IC_50_ = 0.14–0.0045 μM, Table 1) and their limited solubility (<10 μM, Table 1).

**Table 1:** **Summary of the results obtained in the different *in vitro* models for the selected compounds**.

**Compound ID**	**CYP3A4 RI IC_50_ (μM)**	**CYP3A4 TDI K_inac_/KI (min^−1^/μM)**	**Met. stability T_1/2_ (min)**	**NO production IC_50_ (μM)**	**Solubility Range**	
					**Lower bound**	**Upper bound**
Miconazole	0.14	N.A.	N.A.	7.35	8.62	34.48
Clotrimazole	4.51E-3	N.A.	N.A.	7.21	2.59	10.35
Ketoconazole	3.32E-2	N.A.	N.A.	>10	2.59	10.35
Erythromycin	3.21	9.45E-2/0.15 (6.39E-1)	21.66	>250	>86	>86
Roxythromycin	6.41	4.29E-2/0.29 (1.47E-2)	38.08	>250	>86	>86
Claritromycin	4.03	3.99E-2/2.68 (1.49E-2)	50.59	>62.52	>86	>86
Azithromycin	16.1	8.23E-2/7.35 (1.11E-2)	>60	>250	>86	>86
Ciclosporin A	N.A.	N.A.	N.A.	1.16	N.A.	N.A.
Dexamethasone	N.A.	N.A.	N.A.	7.21E-3	N.A.	N.A.
Verapamil	N.A.	0.12/0.79	N.A.	N.A.	N.A.	N.A.

*RI, Reversible inhibition; TDI, Time dependent inhibition; K_inact_, Rate of enzyme inactivation for time dependent inhibitors; KI, Inhibition constant for time dependent inhibitors; T_1/2_, Half-life; NO, Nitric oxide*.

**Figure 1:**
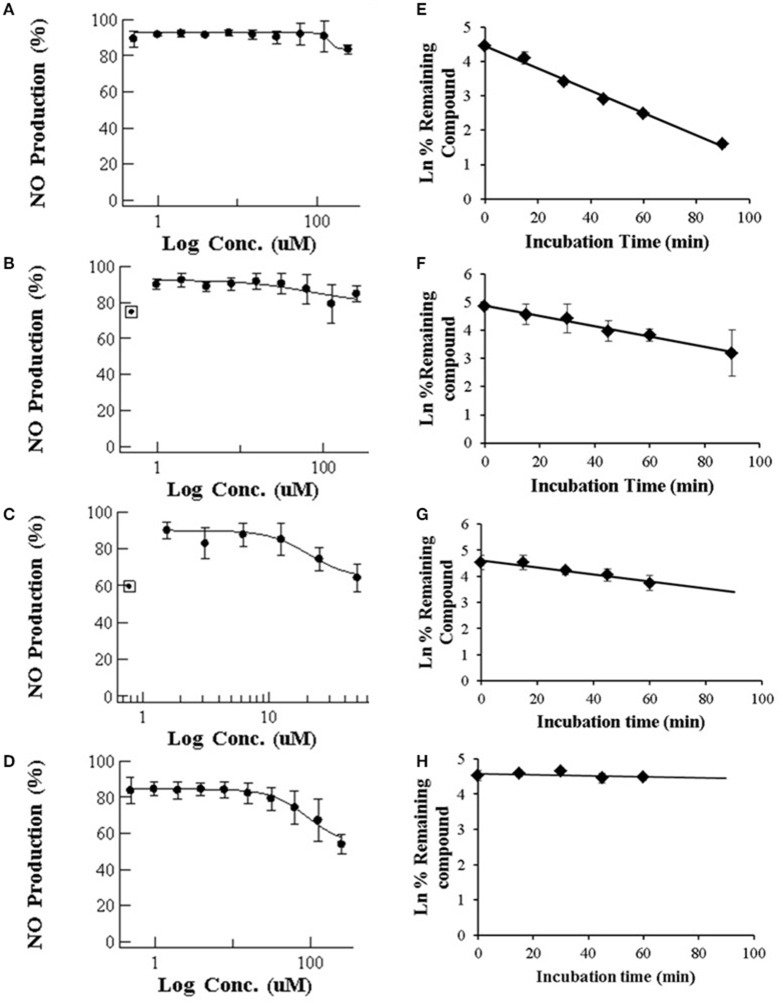
**(Left)** Dose-dependent effects of macrolide antibiotics on the release of NO from LPS-stimulated murine macrophages. The cells were incubated for 24 h with various concentrations of ERY **(A)**, RXT **(B)**, CLT **(C)**, and AZT **(D)**. Max doses (250 μM for ERY, RXT and AZT and 62.5 μM for CLT) were adjusted to achieve a cell viability ≥ 80%. Values on the vertical axe represent the percentage of control of NO formation in the culture medium after addition of LPS. **(Right)** Human *in vitro* liver microsome stabilities (*n* = 3 per time point) for ERY **(E)**, RXT **(F)**, CLT **(G)**, and AZT **(H)**. Data are means ± STDEV; *n* = 6 for each concentration and time point.

Our results for macrolides in LPS stimulated RAW 264.7 cells in contrast with those from primary cultures, showed IC_50_ values higher that the highest noncytotoxic dose (Table 1). Only AZT and CLT had the ability to weakly inhibit the production of NO at the highest noncytotoxic dose (53.82% of control at 250 μM and 85.2% at 62.5 μM, respectively, as shown in Figure 1). In order to check the extent of these interactions, we tested the CYP3A4 inhibitory potency and the solubility range of these macrolide members using NIVA (Table 1). As a result, the CYP3A4 activity was inhibited but to a lesser extent than with imidazole derivatives. Specifically ERY, ROX and CLT displayed, among them, very similar IC_50_ values, in the range of moderate inhibitors (from 1 to 10 μM) whereas AZT behaved as a weak inhibitor (IC_50_ > 10 μM, Table 1). In terms of solubility, macrolide compounds presented solubility limit values >86 μM (Table 1).

### *In vitro* kinetics of CYP3A4 inhibition by macrolides

**Figure 2:**
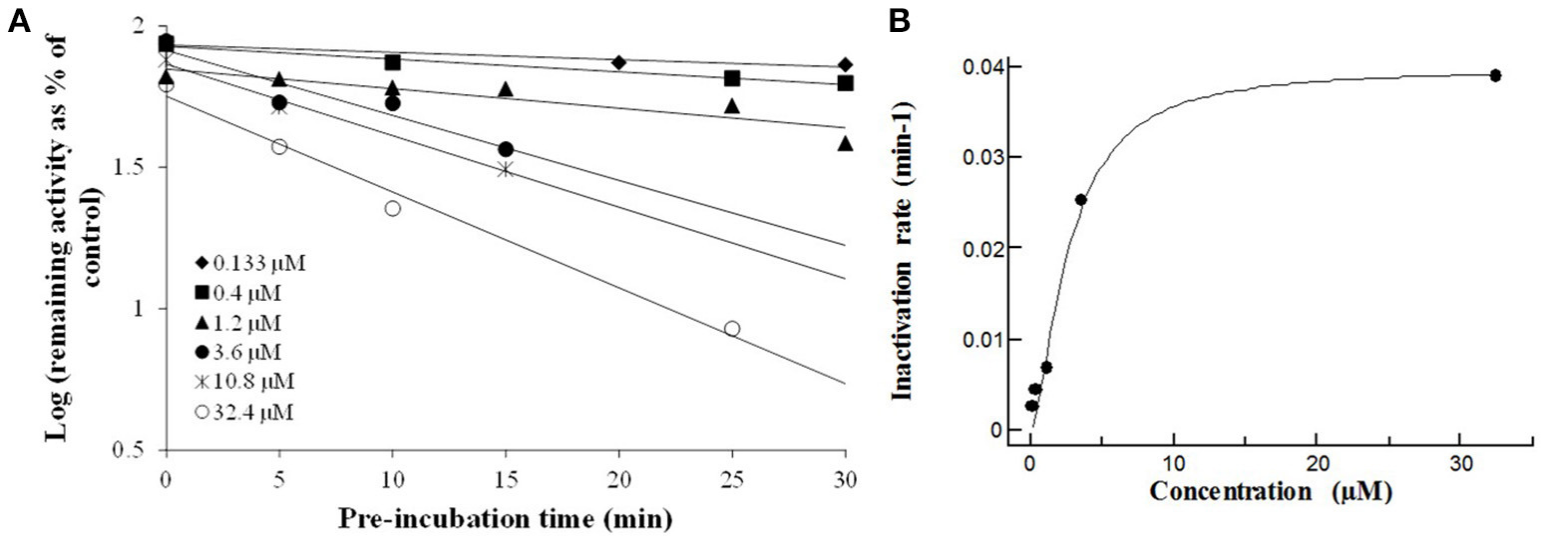
**The macrolides CYP3A4 time dependent inhibition: Compounds were evaluated by pre-incubating a range of 6 test compound concentrations to achieve a scale from no inactivation to maximal inactivation, for 6 differing pre-incubation times with human rCYP3A4 and NADPH**. Following the pre-incubation, an aliquot of the pre-incubation mixture is diluted with buffer containing a specific cytochrome P450 probe substrate (7-HFC) for a specific incubation time (data are singletons for each time and concentration point). **(A)** Inactivation plot (natural logarithm of the % remaining activity vs. pre-incubation time) for CLT. The least-squares regression analysis enables to determine the negative slope of the logarithm of the % remaining activity vs. pre-incubation time. **(B)** The non-linear regression analysis of the negative slopes against inhibitor concentration enables K_inact_ and KI to be calculated. The K_inact_ is the maximal rate of enzyme inactivation at a saturating concentration of inhibitor and KI is the concentration of inhibitor which gives half the maximal rate of inactivation. Similar time- and concentration-dependent inhibition profiles were observed ERY, RXT, and AZT (data not shown).

In order to establish the *in vitro* macrolides kinetic constants of CYP3A inhibition, ERY, CLT, RXT, and AZT were estimated by varying the time of pre-incubation and the concentration. The two major kinetic parameters that characterize time-dependent inhibition interactions (TDIs) are the maximal inactivation rate constant (K_inact_) and the inhibitor concentration leading to 50% of enzyme inactivation (KI), see Figure 2. The K_inact_/KI ratio is commonly taken as an indicator of the *in vitro* potency of a mechanism-based inhibitor. The derived kinetic constants from the inactivation experiments are presented in Table 1. Values of K_inact_ and KI were in the range of 0.0399–0.0945 min^−1^ and of 0.146–7.349 μM, respectively, indicating that all 4 macrolides inhibited in a time dependent manner the BFC conversion into 7-HFC in CYP3A4 human recombinant microsomes with an inactivation potency (K_inact_/KI ratio) in the following order: ERY > RXT > CLT > AZT.

### Macrolide *in vitro* metabolic stability

Following incubation in HLM, all four macrolides displayed different half-life (T_1/2_) values in the range from 21 to >60 min. This value increased in the order ERY < RXT < CLT < AZT (Table 1 and Figure 1). These results indicate that the biotransformation rate of ERY and, consequently, the metabolite formation is about double that of RXT and CLT while non-detectable biotransformation was observed for AZT at the referred incubation times.

### RXT metabolite profile in HLM incubations

**Figure 3:**
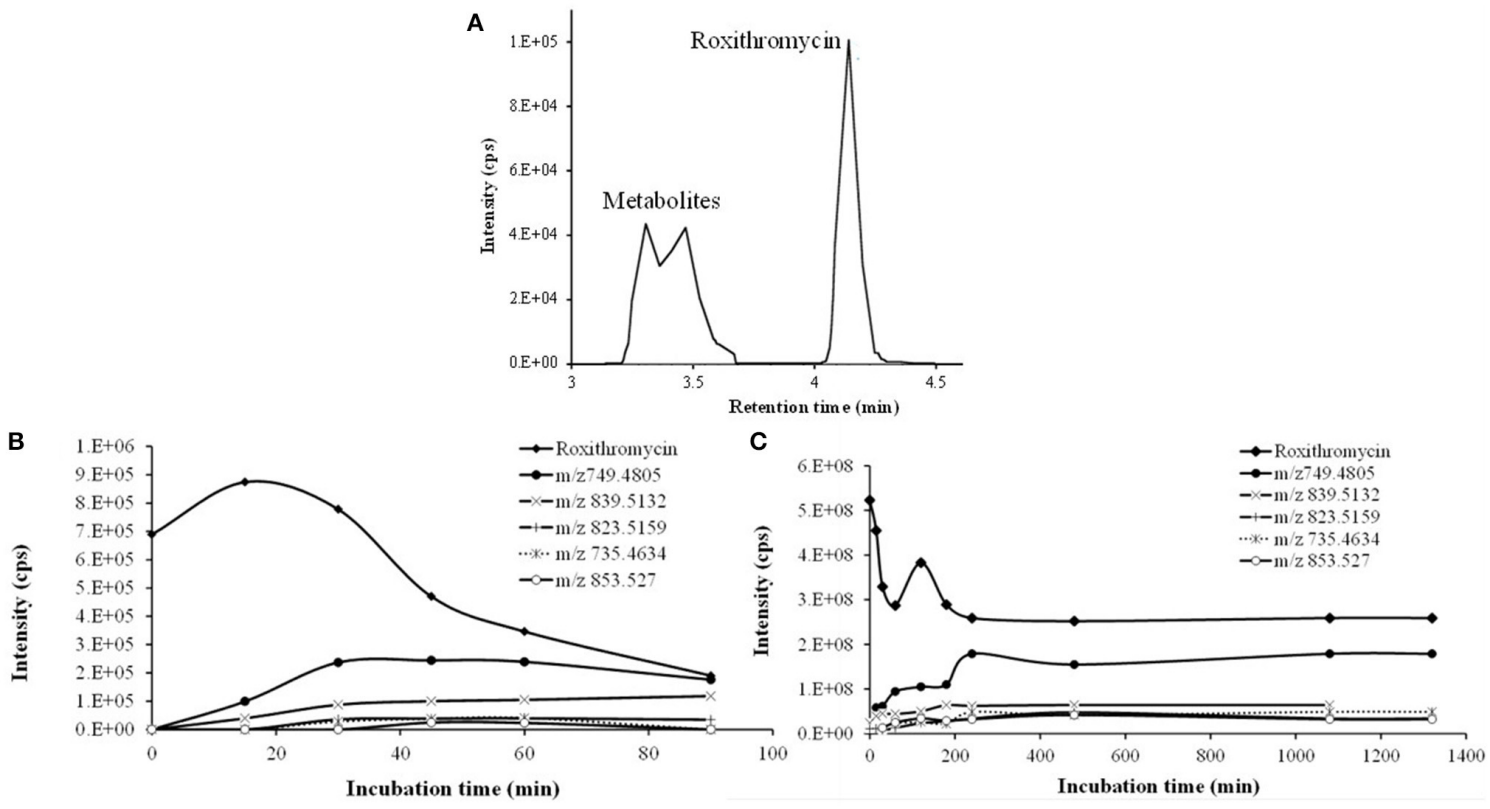
**Time course of RXT incubations with HLM. (A)** Representative combined extracted ion chromatogram (XIC) of RXT and its metabolites at 30 min incubation time. The main peak at 4.12 min was identified as RXT by direct comparison with the authentic standard. The rest of peaks correspond to RXT metabolites, which were not observed in the control samples. **(B)** Time course of RXT at small scale incubation. **(C)** Time course of RXT at large scale incubation (data are singletons for each incubation time).

The characterization of the *in vitro* metabolism of RXT was achieved from the LC-HRMS data obtained in the metabolic stability incubations. As a result, in addition to unchanged RXT, a total of 5 stable potential metabolites were found (Table 2 and Figure 3A). Among them, besides the metabolites of RXT identified by other investigators (Zhong et al., 2000) (ERY oxime at m/z 749, demethylated ERY oxime at m/z 735 and N-mono-demethylated RXT at m/z 823), we identified another 2 additional stable biotransformation products (hydroxylated RXT at m/z 853 and demethylated and hydroxylated RXT at m/z 839). The molecular structure elucidation of the potential metabolites was carried out by fragment interpretation of CID spectral data with the assistance of chromatographic behaviors and basic rules of drug metabolism. This is discussed in the following sections. The relative content of each metabolite was estimated with reference to RXT on the basis of peak area of their [M+H]^+^ ion. According to this criteria, metabolite M1 (ERY oxime at m/z 749) is the main biotransformation product (20%) of RXT in HLM incubations. The relative contents for the rest of metabolites (M2–M5) ranged from 7 to 2% (Table 2) of RXT peak area as shown in Figure 3B. The RXT metabolite profiling was monitored at different incubation times, obtaining the same main metabolites with very similar formation rates to those obtained in small scale incubations (Figure 3C).

**Table 2:** **Summary of key LC/HRMS data of *in vitro* metabolites after RXT incubation with HLM**.

**Peak ID**	**Description**	**Formula**	**m/z**	**Mass error (ppm)**	**R.T. (min)**	**Peak area**	**Area (%)**	**Score (%)**
	RXT (Parent)	C_41_H_76_N_2_O_15_	837.5331	1.5	4.12	7.78E+05	65	93.9
M1	ERY oxime	C_37_H_68_N_2_O_13_	749.4805	1.5	3.64	2.37E+05	20	93.7
M2	Demethylatied + Hydroxylated RXT	C_40_H_74_N_2_O_16_	839.5118	0.8	3.52	8.74E+04	7	86.1
M3	N-mono-demethylated RXT	C_40_H_74_N_2_O_15_	823.5169	0.9	3.68	3.57E+04	3	95.5
M4	Mono hydroxylated RXT	C_41_H_76_N_2_O_16_	853.527	0.3	3.74	2.46E+04	2	79.3
M5	Demethylated ERY oxime	C_36_H_66_N_2_O_13_	735.4638	0.1	3.56	2.82E+04	2	81.5

### Isolation and structure elucidation of roxithromycin metabolites

**Figure 4:**
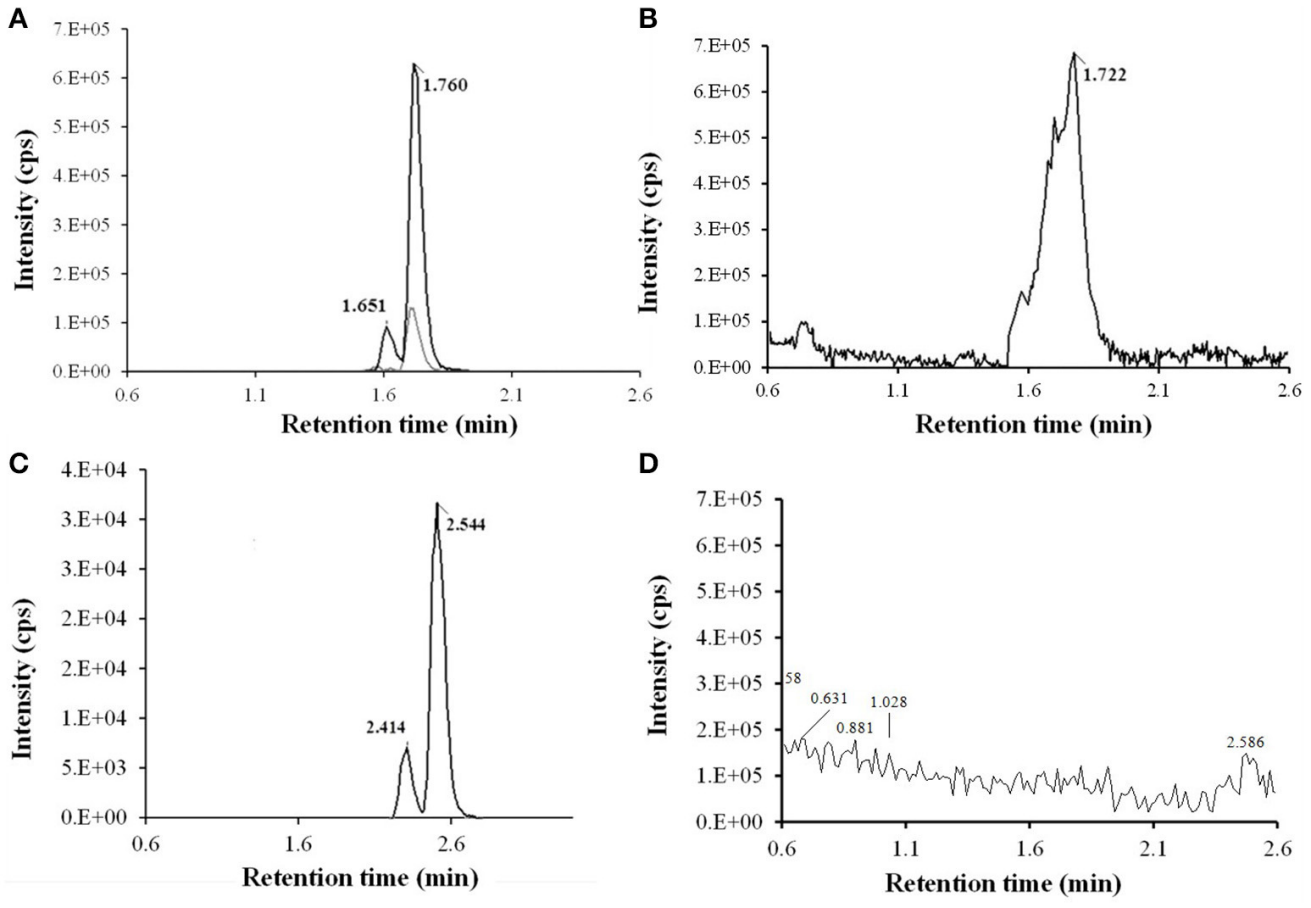
**LC-HRMS chromatograms of RXT metabolite isolation using fast gradient separation. (A)** Extracted ion chromatogram (XIC) of semi-preparative fraction (at elution time 12.5) for expected RXT metabolites showed 3 main peaks: 2 peaks at m/z 749.4805 (in black color line) at 1.65 and 1.76 min corresponding to (Z)-ERY-oxime and (E)-ERY-oxime (M1), respectively and 1 peak at m/z 735.4638 (in light gray color line) and 1.76 min, corresponding to metabolite M5. Although very weakly the corresponding (Z) isomer was also observed at 1.65 min. **(B)** TIC (50-1000 Da) of semi-preparative fraction (at elution time 12.5. **(C)** TIC (50-1000 Da) of semi-preparative fraction from blank incubation at 12.5 min elution time. **(D)** XIC of RXT reference substance showed 2 peak at m/z 837.5331 corresponding to RXT (2.54 min) and its Z-stereoisomer (2.41 min), which were not observed in the metabolite enriched fraction, ensuring the absence of parental drug.

**Figure 5:**
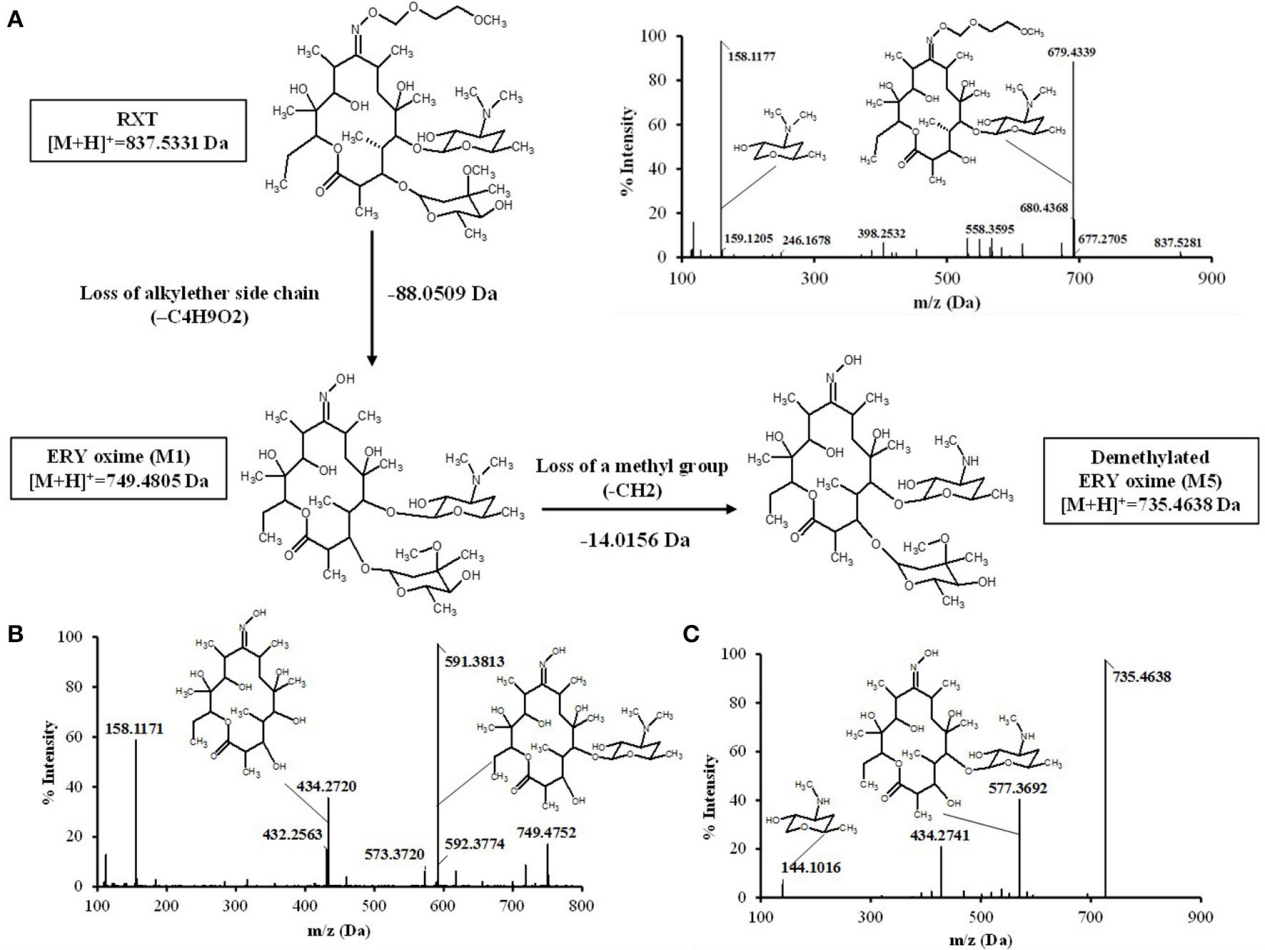
**Proposed biotransformation route from RXT to metabolites ERY oxime (M1) and Demetylated ERY oxyme in HLM incubations**. The loss of 88 Da from RXT at [M+H]^+^ = 837.5331 to M1 at [M+H]^+^ = 749.4805 Da is fully compatible with the loss of the alkylether side chain. Fragment ion analysis of MS/MS spectrum from RXT **(A)** and M1 **(B)** revealed an analog loss of 88 Da from m/z = 679 Da in RXT to m/z = 591 Da in M1, confirming the site of alkylether chain at the lactone ring. The ion at m/z 591was 158 Da lower (loss of cladinose) than the precursor ion (m/z 749), whereas the ion at m/z 434 was subsequently 157 Da lower (loss of desosamine) than the former ion. Based on these data, M1 was identified as ERY-oxime. The difference of 14 Da between M1 at [M+H]^+^ = 749.4805 Da and M5 at [M+H]^+^ = 735.4638 Da indicated the loss of a methyl group in the biotransformation. The MS/MS spectra of m/z 735 Da **(C)** gave fragment ions at m/z 577, 434, and 144 Da. The ions at m/z 434 Da indicated the lactone ring was unaltered. The loss of the cladinose moiety produced the peak at m/z 577 Da, suggesting that the demethylation took place at the desosamine that was confirmed by the peak at m/z 144 Da. Based on these data M5 identified as demethylated ERY oxime.

After SPE and LLE of RXT large scale incubations for isolation of its main metabolites, semi-preparative HPLC chromatography was used to obtained fractions containing RXT metabolites. After several steps of isolation and purification guided by the extracted ion chromatogram (XIC) of previously observed metabolites, we achieved 1 mg of highly enriched fraction (at elution time of 12.5 min) in 2 main metabolic products (M1&M5, Figure 4A). As previously described, purity was assessed by comparison of the detector response to the metabolites in the incubation matrix with the response to a blank incubation matrix at the same elution time (Figures 4B,C) (Espina et al., 2009; Walker et al., 2011). The assessment by total ion chromatogram (TIC) delivered a purity of 93% for the RXT metabolites contained in that fraction, which is in line with the general purity target for library compounds for activity screening (Kassel, 2007). The identification of these metabolites was achieved by comparative fragment ion interpretation with the commercial reference substance of parent drug (RXT, chemically designated as (*E*)-ERY-9-[O-[(2-methoxyethoxy)methyl]oxime]) and previously results from other investigators using LC-MS and nuclear magnetic resonance (NMR) (Zhong et al., 2000). Briefly, full scan high resolution mass spectra of RXT reference substance provided 2 chromatographic peaks with a 5-fold intensity ratio, at identical m/z 837 but different retention times (2.41 and 2.54 min, see Figure 4D). Both chromatographic peaks displayed the same MS/MS fragment ions at m/z 679, 558, 540, and 158 (Figure 5A), suggesting they were stereoisomers with tertiary amino groups in their structure. They were thus identified as RXT and its Z-stereoisomer. For metabolite M1, we also observed 2 chromatographic peaks at retention times of 1.65 and 1.76 min, displaying a 7-fold intensity ratio (Figure 4B). They showed identical protonated molecular ions [M+H]^+^ at m/z 749 as well as identical MS/MS fragment ions, indicating they were also stereoisomers. Their protonated molecular ions (m/z 749) were 88 Da lower (characteristic loss of the alkylether side chain) than that of the parent drug (m/z 837), indicating they were *O*-dealkylated metabolites. The MS/MS spectra of both peaks (parent ion at m/z 749) gave fragment ions at m/z 591, 434, and 158 Da (Figure 5B). The ion at m/z 591was 158 Da lower (loss of cladinose) than that of the precursor ion (m/z 749), whereas the ion at m/z 434 was subsequently 157 Da lower (loss of desosamine) than the former ion. Based on these data, they were identified as (*Z*)- and (*E*)-ERY-oxime, respectively. For metabolite M5 the above described (*Z*) and (*E*) isomers distribution was also observed at 1.65 and 1.76 min (Figure 4B). Their protonated molecular ions (m/z 735) were 102 Da lower than that of the parent drug, indicating a cleavage of the alkylether side chain (88 Da), followed by the loss of a methyl group. The MS/MS spectra of m/z 735 gave ions at m/z 577, 434, and 144 (Figure 5C). The diagnostic ions at m/z 434 indicated that the 14-member lactone ring nucleus was unaltered. The loss of the cladinose moiety produced the peak at m/z 577, suggesting that the demethylation was carried out on the desosamine that was confirmed by the peak at m/z 144 (Figure 5C). Based on these data these peaks were identified as *N*-monodemethylated derivatives of (*Z*)- and (*E*)-ERY-oxime, respectively. The stereochemical aspects of RXT and its metabolites have been extensively described in previous works using LC-MS in combination with NMR and our results are consistent with them (Zhong et al., 2000). Therefore, 2 RXT major metabolites (M1–M5) and their corresponding (*Z*)- isomers were isolated from the rest of the components of microsomal incubations in a semi-preparative HPLC fraction which yielded a dry weight of 1 mg. Since our main objective was to clarify the implication of RXT metabolites in inactivation of NO production and not activity ascription, we considered that isolation of single metabolites was not critical, being much more important the separation from parent drug (RXT) and main matrix components. Regarding the relative composition we could conclude from XICs that metabolite M1and M5 represents 78% [68 and 10% for (*E*) and (*Z*)-isomers, respectively] and 15% [14 and 1% for (*E*) and (*Z*)-isomers, respectively] of the total dry amount, respectively. It is very interesting to notice that the (*E*)-isomer of RXT was reported to be the more potent of the two geometric forms in antibacterial tests and consequently, RXT in clinical use exclusively contains this isomer (Zhong et al., 2000). Hence, we can assume that the observed therapeutically effects are due to the (*E*) isomers of either RXT or its metabolites.

### Biological evaluation of isolated RXT metabolites

**Figure 6:**
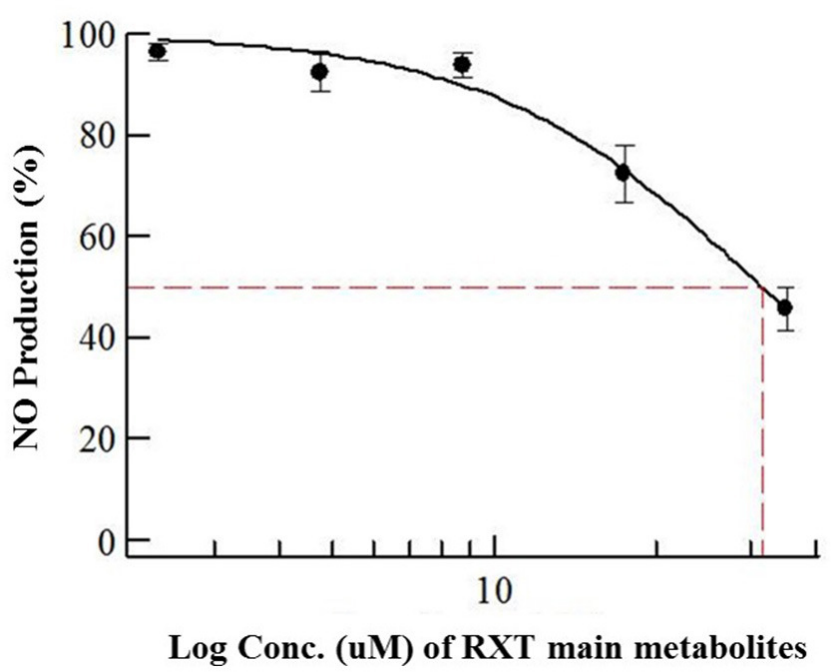
**Dose-dependent effects of RXT metabolites (M1 and M5) on the release of NO from RAW 264.7 mouse macrophages stimulated with LPS**. Data are means ± STDEV from experiments in triplicate (*n* = 3) for each concentration point.

Considering previously reported RXT IC_50_ value (5 μg/mL) in NO production using primary cultures of alveolar rat macrophages, we dissolved the dry amount (1mg) of RXT metabolites (M1, M5 and the corresponding geometric isomers) in 0.133 mL of pure DMSO (Tamaoki et al., 1999; Kohri et al., 2000). After ½ serial dilution in DMSO, the metabolite mixture was 1/5 diluted in water in order to minimize the DMSO content, and it was finally tested (max dose 35 μg/mL) in triplicate using the LPS stimulated RAW 274.6 macrophage model to evaluate its ability to inhibit NO production. The mixture of RXT metabolites had the ability to inhibit NO production to 50% at a concentration of 31.6 μg/mL (Figure 6) while no inactivation at all of NO production was observed for RXT from 0.488 to 250 μM (Figure 1B).

Considering relative composition of the RXT metabolite mixture and the previously discussed isomer activity ascription, it is possible to infer an IC_50_ value of 23.07 and 4.74 μg/mL for M1 and M5, respectively, for inactivation of NO production. These values are in line with the previously reported RXT IC_50_ value (5 μg/mL) in NO production using primary cultures of alveolar rat macrophages.

## Discussion

In order to characterize the involvement of the CYP450 enzymatic system in immunomodulatory properties of the previously described molecules (imidazole derivatives and macrolides), we selected representative members of the 2 families of compounds (ketoconazole, miconazole and clotrimazole from the imidazole derivate family, and ERY, RXT, CLT and AZT from macrolides family) for the assessment of NO production in the LPS stimulated RAW 264.7 macrophage *in vitro* model. Therefore, and taking into consideration that a loss of biotransformation activity has been observed in cultured cells (RAW 264.7) caused by a decrease in CYP450 transcription in comparison with primary cell cultures (alveolar macrophages) or *in vivo* experimental systems, a differential response in NO production should be expected for dual inhibitors depending on the considered experimental model (Rodríguez-Antona et al., 2002; Zarogoulidis et al., 2012). Thus, a compound being intensively metabolized by CYP450 should display a higher inhibition potential over NO production in this *in vitro* model (LPS stimulated RAW 264.7) with reduced CYP450 functionality. On the contrary, a compound producing active metabolites via CYP450 should show a reduced potential to inhibit NO in comparison with other *in vitro* models with regular CYP450 functionality. Our results for imidazole derivatives in NO production when compared with the *in vivo* results reported for related azole compounds (even at higher doses) showing no inhibition of NOS activity, support, this way, our working hypothesis in which the diminished CYP450 activity in RAW 264.7 cells may have some influence on NO production (Dercho, 2015). In this case, the absence of compound depletion may contribute to the moderate IC_50_ values obtained in comparison with the complete lack of inhibition NO production observed using an *in vivo* experimental system. The CYP3A4 inhibition data showed that imidazole derivatives exerted their inhibition ability on CYP3A4 in the soluble range, suggesting that their limited solubility (upper solubility limit from 10.35 to 34 μM) could be related with the moderate ability to *in vitro* inhibit NOS and even with the lack of *in vivo* response. Therefore, it is reasonable to expect that the actual cytoplasmatic concentration of imidazole derivatives in RAW 264.7 cells might be much lower than anticipated and hence, might show artificially low inhibition on NO production. In fact, the IC_50_ values (0.14 μM) obtained by other investigators for ketoconazole on CYP3A4 activity using cryopreserved hepatocytes were 4-fold higher than those got in our experiment with recombinant CYP3A4 enzymes (0.033 μM) (Moeller et al., 2012).

In the case of macrolides, other investigators have demonstrated that these compounds (ERY, RXT and CLT) could inhibit the production of NO in either primary cultures of stimulated rat pulmonary alveolar macrophages or stimulated animal models model by down-regulation of NOS gene expression (Hukkanen, 2000; Zhong et al., 2000; Kanoh and Rubin, 2010). However the therapeutic mode of action and the biotransformation of macrolides are not well understood and many questions remain unanswered, such as the role of NOS inhibition or the implication of their main metabolites in the therapeutic response. Our *in vitro* results showing a lack of potential activity to inhibit NO production in comparison with other experimental systems with drug metabolic competence also supported our previously exposed working hypothesis and it points toward the critical role of macrolide biotransformation via CYP450 in the inactivation of NO production. Our data from CYP3A4 reversible inhibition confirm the differential ability of these compounds to inhibit CYP3A4 activity in a concentration dependent manner which interestingly correlates reversely with their potential to inhibit NO production in LPS stimulated RAW 264.7 cells (Figure 1, left and Table 1). The results from our macrolide KS experiments enabled to suppress the effect of solubility issues in the observed results.

Macrolide antibiotics have been previously described as mechanism-based inactivators of CYP3A enzymes that exhibit varying degrees of inhibitory potency. Mechanism-based inhibition often involves the metabolism of an inhibitor by CYP3A4 to a reactive metabolite, which inactivates the catalyzing enzyme in a concentration and time-dependent manner. The interaction between the inactivating species and the enzyme can either be covalent or non-covalent, involving binding to protein or heme moiety, respectively (Polasek and Miners, 2006). From our results in CYP3A4 time dependent inhibition experiments, we can assert that for these macrolide antibiotics, CYP3A4 is responsible for the production of active metabolites which might be central to inhibit NO production. Hence, important information can be drawn:

First, although ERY, RXT, and CLT got very similar IC_50_ values from CYP3A4 reversible inhibition, we can observe a clear shift in K_inact_/KI ratio from ERY and RXT (0.64 and 0.14 min^−1^/μM, respectively) to CLT and AZT (0.014 and 0.010 min^−1^/μM, respectively). These results indicate that product metabolites from ERY and RXT are much more potent inhibitors than those produced from CLT and AZT which is on line with the differential CYP3A4inhibition potency observed among the macrolides in the clinic (Polasek and Miners, 2006).

Second, the inability of RAW 264.7 cells to produce these active metabolites might partially explain the lack NO inhibition observed for macrolides in this cell line.

The *in vitro* metabolic stability, expressed as intrinsic T_1/2_ of selected macrolide antibiotics, was investigated in HLM incubations. Pointing in the same direction as TDI results, these data also showed that compounds undergoing more intense biotransformation (ERY and RXT) did not affect NO production in RAW 267.4 cell lines (Figures 1A,B,E,F) while those displaying higher microsomal stability (CLT and AZT) exert a certain degree of inactivation in NO production (Figures 1C,D,G,H). This fact suggests that the reduced expression CYP450 isoform and the subsequent lack of reactive metabolites might be the cause of the inability of ERY and RXT to inhibit NO production in RAW 267.4 cell lines. Although higher quantity and distribution of metabolites is expected for ERY based on our stability results, the more abundant previous information related to *in vivo* and *in vitro* metabolism as well as the increasing interest aroused by RXT's anti-inflammatory and immunomodulatory properties in clinical practice, led us to select this macrolide to explore its *in vitro* metabolic profile in detail (Yamazaki and Shimada, 1998; Tamaoki et al., 1999). For RXT, as stated in the result section, main biotransformation took place in N-oxime ether side chain attached to the lactone ring, rendering an oxime functional group. This metabolism feature leads to a higher exposition of imine moiety (carbon–nitrogen double bond) which is also observed in imidazole derivatives and suggests that this biotransformation and the corresponding metabolites may have a key role in differential clinical effects observed for RXT (Zarogoulidis et al., 2012).

Authentic standards of drug metabolites are valuable reagents for carrying out experiments aimed toward understanding the disposition, efficacy, and safety of drugs. However, obtaining metabolite standards by conventional organic synthesis can frequently be challenging, expensive, and they can take weeks to months to prepare. As an alternative approach, the biological generation and isolation of metabolites from source material obtained from *in vivo* or *in vitro* studies has been proved to useful for the production of isolates in the nanomole range. Metabolites obtained using these approaches can be used to test target activity, they can be used as authentic standards for quantitative bioanalysis of *in vivo* and *in vitro* samples by HPLC-MS, and they can also be used in other *in vitro* drug metabolism experiment. In order to isolate main microsomal metabolites of RXT with the objective of assessing their involvement of NO modulation, we carried out a large scale incubation as described in methods and materials section. Therefore, we can assert from our results that the metabolites of RXT (M1&M5) demonstrated significant activity as inhibitors of NO production, affording better activity than the parent drug in LPS stimulated RAW 274.6 macrophage cell lines.

Although our results showed that quite high concentrations of RXT metabolites are required to inhibit NOS activity, the relevance of this *in vitro* observations should be taken into consideration since there are clear and compelling data demonstrating their nonantimicrobial properties of macrolide, such as their ability to decrease the hypersecretion of key regulators of the inflammatory response in cell culture, in animal models of disease, and in persons with chronic inflammatory pulmonary diseases (Kanoh and Rubin, 2010). It has been proven that macrolides can decrease mucus hypersecretion both *in vitro* (Okamoto et al., 1999) and *in vivo* (Tamaoki et al., 1995; Rubin et al., 1997). In particular, physiological concentrations of ERY and CLT inhibit IL-8 mRNA and protein in bronchial epithelial cells from healthy subjects and those with chronic inflammatory airway diseases (Takizawa et al., 1997). ERY at a concentration of 10 μg/ml inhibited IL-6, IL-8, and soluble intercellular adhesion molecule 1 secretion from human bronchial epithelial cells stimulated by endotoxin (Khair et al., 1995). ERY decreased production of tumor necrosis factor Alpha and IL-6 in human whole blood stimulated with heat-killed *Streptococcus pneumoniae* (Schultz et al., 1998). Similar effects have been observed using RXT, which suppressed the production of IL-6, IL-8 in the human epithelial cell line Bet-1A stimulated with IL-1α (Kawasaki et al., 1998). These immunomodulatory activities appear to be polymodal, so macrolides accumulate within cells, suggesting that they may associate with receptors or carriers responsible for the regulation of cell cycle and immunity (Kanoh and Rubin, 2010). The characteristics of the clinical nonantimicrobial properties of macrolide therapy include effective doses that are much lower than the minimal inhibitory concentration (i.e., low-dose macrolide therapy) (Nagai et al., 1991; Fujii et al., 1995; Kudoh et al., 1998; Shitrit et al., 2005).

As general mechanism of *in vivo* inhibition, we propose two compatible approaches. First, as previously described alveolar macrophages represent a clear evidence for co-expression of these enzymes (CYP450s and NOS) and this can be consider as a common fact to other cell types in primary sites of exposure for chemical toxicants such as hepatic macrophages which present an expression pattern of CYP450 specifically adapted for their major role in the protection of the organism (Rubin et al., 1997; Okamoto et al., 1999; Hukkanen et al., 2002). Therefore it makes sense to expect that compound biotransformation and NOS inhibition might take place in the same cell. Second and in parallel, hepatic metabolites might be cleared to plasma and distributed systemically to immune system cells and epithelial tissues in which might inhibit NOS activity.

## Conclusions

This study represents an initial comprehensive characterization of CYP450 involvement in immunomodulatory response in the macrophage cell lines. Even though the assessment of CYP450 protein levels, inter-species metabolic differences and activity measurements will be necessary to further validate and confirm our results, this study suggests that the metabolites of RXT might be responsible for some of the observed pharmacological activity of RXT, highlighting the role of CYP450 activity in the development of the pharmacological immunomodulation, especially for those compounds undergoing time-dependent biotransformations through this enzymatic system.

## Author contributions

Authors make substantial contributions to conception and design, and/or acquisition of data, and/or analysis and interpretation of data; JP, CD, NV, JG, FV. Authors participate in drafting the article or revising it critically for important intellectual content; FA, AR, Nd, MR, OG. Authors give final approval of the version to be submitted and any revised version; JG, FV.

### Conflict of interest statement

The authors declare that the research was conducted in the absence of any commercial or financial relationships that could be construed as a potential conflict of interest. The reviewer AM and handling Editor declared their shared affiliation, and the handling Editor states that the process nevertheless met the standards of a fair and objective review.

## Abbreviations

AcN: Acetonitrile
AZT: Azithromycin
BFC: 7-Benzyloxy-4-(trifluoromethyl) coumarin
CID: Collision induced dissociation
CLT: Clarithromycin
CYP3A4: Cytochrome P450 isoform 3A4
CYP450: cytochrome P450
DMSO: Dimethyl sulfoxide
ERY: Erythromycin
HLM: Human liver microsomes
HPLC: High Performance liquid chromatography
iNOS: Inducible nitric oxide synthase
IL: Interleukin
Kinact: Rate of enzyme inactivation for time dependent inhibitors
KI: Inhibition constant for time dependent inhibitors
KS: Kinetic solubility
LC-HRMS: Liquid chromatography coupled to high resolution mass spectrometry
LC-MS: Liquid chromatography coupled to mass spectrometry
LLE: Liquid-liquid extraction
LPS: Lipopolysaccharide
MS: Mass spectrometry
MS/MS: Tandem mass spectrometry
N.A.: Not Applicable
NADPH: Reduced nicotinamide adenine dinucleotide phosphate
NIVA-CYPI-KS: Novel *in vitro* approach to simultaneously assess CYP450 inhibition and KS solubility
NMR: Nuclear magnetic resonance
NOS: Nitric-oxide synthase
NO: Nitric oxide
QTOF: Quadrupole time of flight
RAW 264.7: Macrophage cell line; Abelson murine leukemia virus trans-formed
RXT: Roxythromycin
STDVE: Standard deviation
SPE: Solid phase extraction
T1/2: Half-life
TDI: Time dependent inhibition
TIC: Total ion chromatogram
UGT: Uridine diphosphate glucuronosyltransferase
XIC: Extracted ion chromatogram.
